# Supplementary material for: Using mobile phone data to reveal risk flow networks underlying the HIV epidemic in Namibia
Source: Nat Commun. 2021 May 14;12:2837. doi: 10.1038/s41467-021-23051-w (PMC8121904; doi:10.1038/s41467-021-23051-w)
Supplement: Supplementary file 1 — Supplementary Information [file 41467_2021_23051_MOESM1_ESM.pdf]

Supplementary Information

Using mobile phone data to reveal risk flow networks underlying the HIV epidemic in Namibia

Authors: Eugenio Valdano[1], Justin T. Okano[1], Vittoria Colizza[2], Honore K. Mitonga[3], Sally Blower[1]*

Affiliations:

[1]Center for Biomedical Modeling, The Semel Institute for Neuroscience and Human Behavior, David Geffen School of Medicine, University of California, Los Angeles, CA 90095.

[2]INSERM, Sorbonne Université, Institut Pierre Louis d'Epidémiologie et de Santé Publique, IPLESP, Paris, France.

[3]Department of Epidemiology and Biostatistics, School of Public Health, University of Namibia, Windhoek, Namibia.

*Corresponding author: sblower@mednet.ucla.edu

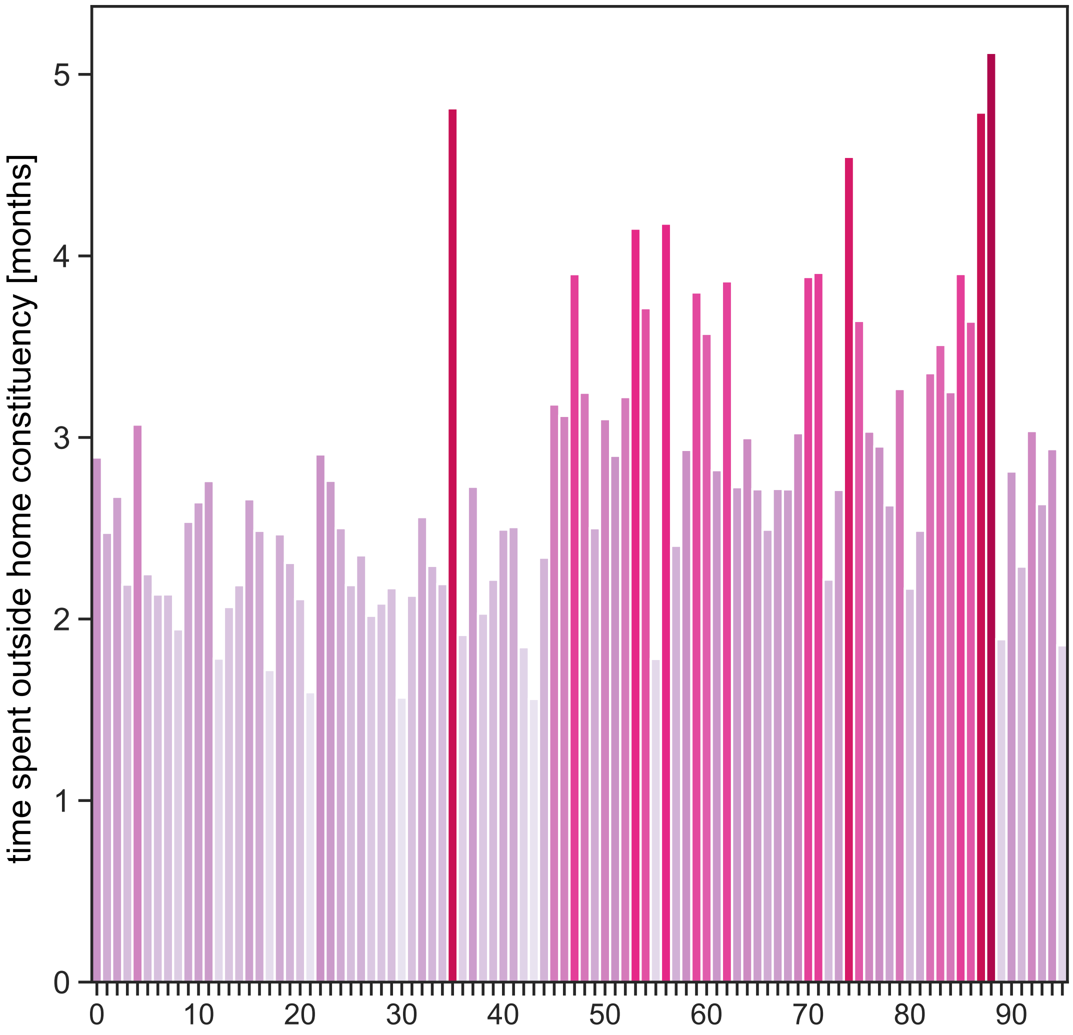

Supplementary Figure 1: Histogram showing the proportion of time (between October 2010 and September 2011) that an average resident spent outside their home constituency. This histogram was derived from the CDR dataset. The colors match those in the map shown in Fig. 2a. The constituencies (shown on the x-axis) are ordered in terms of increasing prevalence of HIV in women, from 6% to 39%. The key code for constituencies is given in Supplementary Table 4.

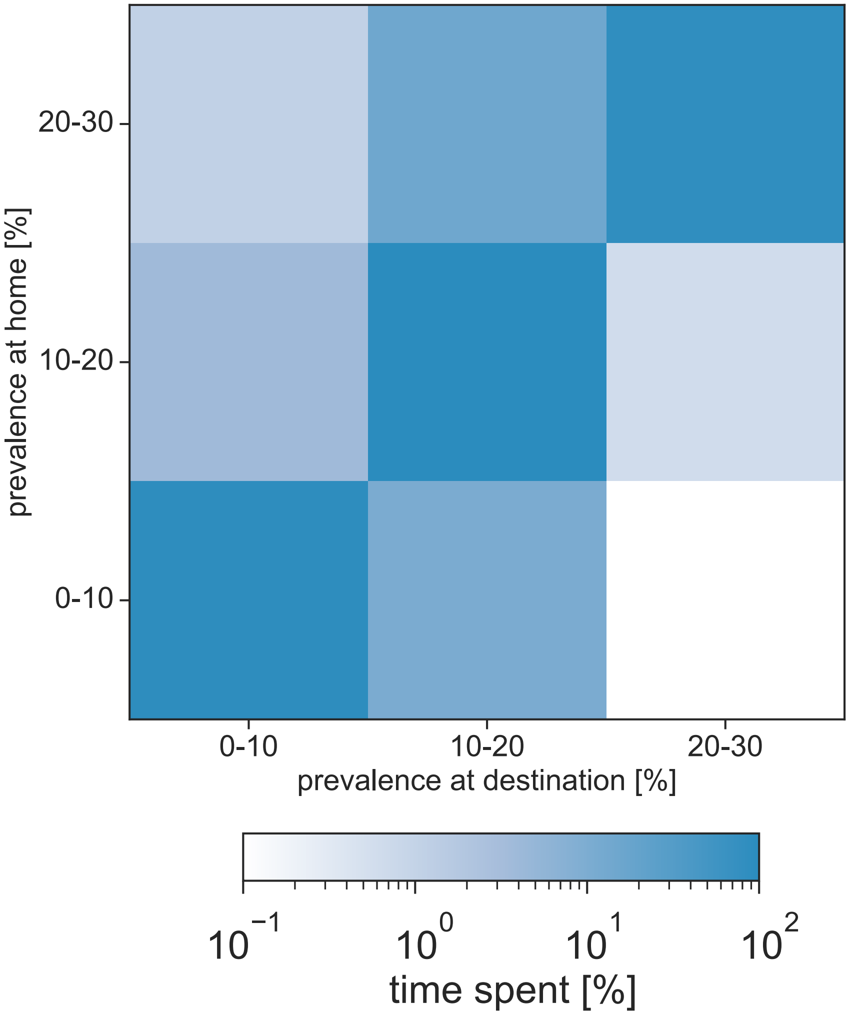

Supplementary Figure 2: Spatial relationships for mobility and HIV prevalence. Matrix showing the proportion of time (over a year) that women in Namibia spent in each prevalence "class", as a function of the prevalence class of their home constituency. Prevalence refers to prevalence in men. The matrix is color-coded to show the fraction of time spent in the destination constituency. A logarithmic scale is used, ranging from 0.1% to 100%.

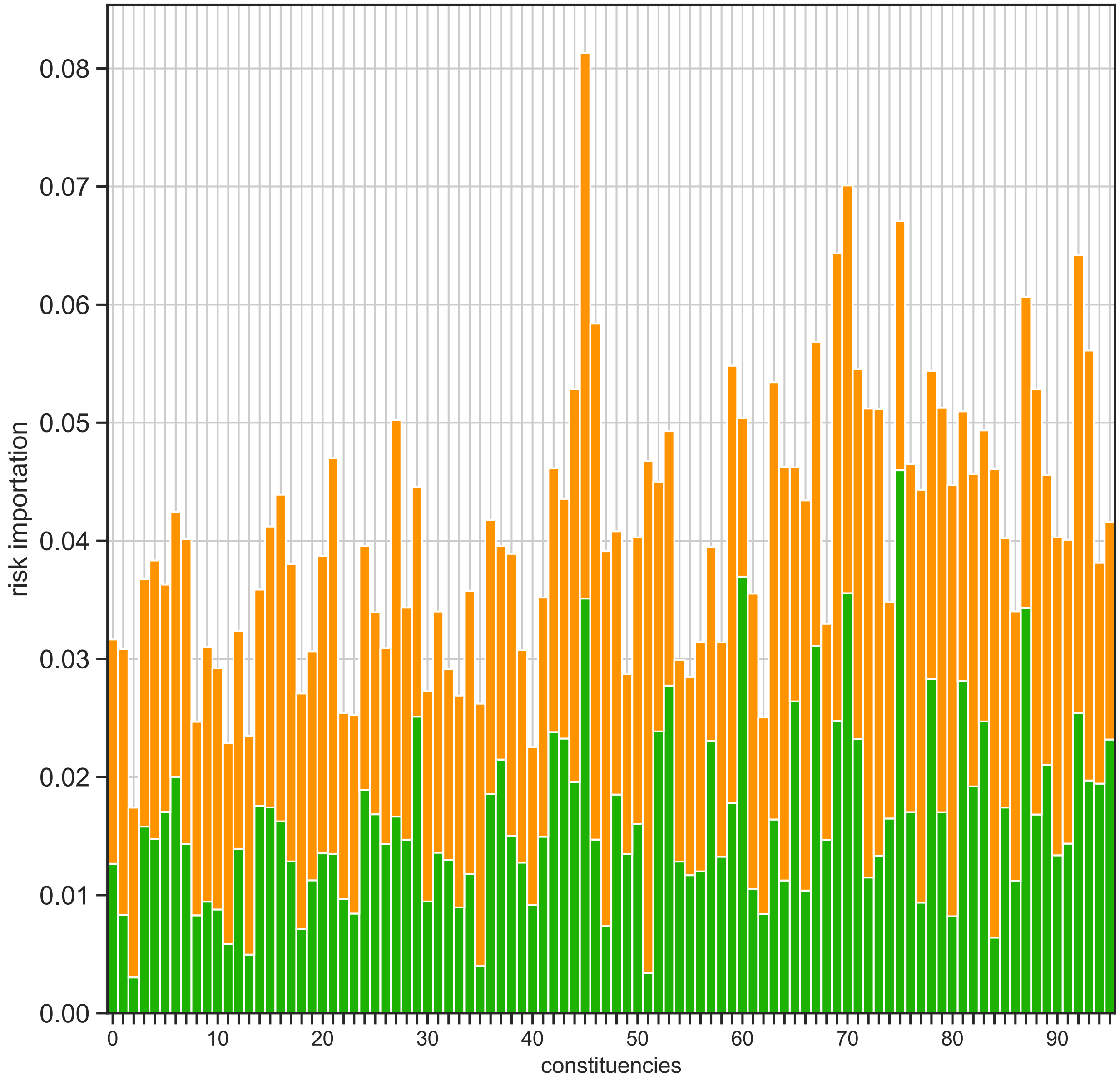

Supplementary Figure 3: Importation of risk for women. Histogram showing the importation of risk for women into each constituency. The y-axis shows the value for imported risk; a mathematical definition of imported risk is given in equation (5). The numbers on the x-axis refer to specific constituencies; the key code is given in Supplementary Table 3. Constituencies are ordered by increasing HIV prevalence in men: from 0% to 24%. Orange data show the risk for women that was imported into each constituency due to travel by their uninfected female residents. Green data show the risk for women that was imported into each constituency due to visits from HIV-infected men who lived in other constituencies. The stacked value represents the total amount of imported risk.

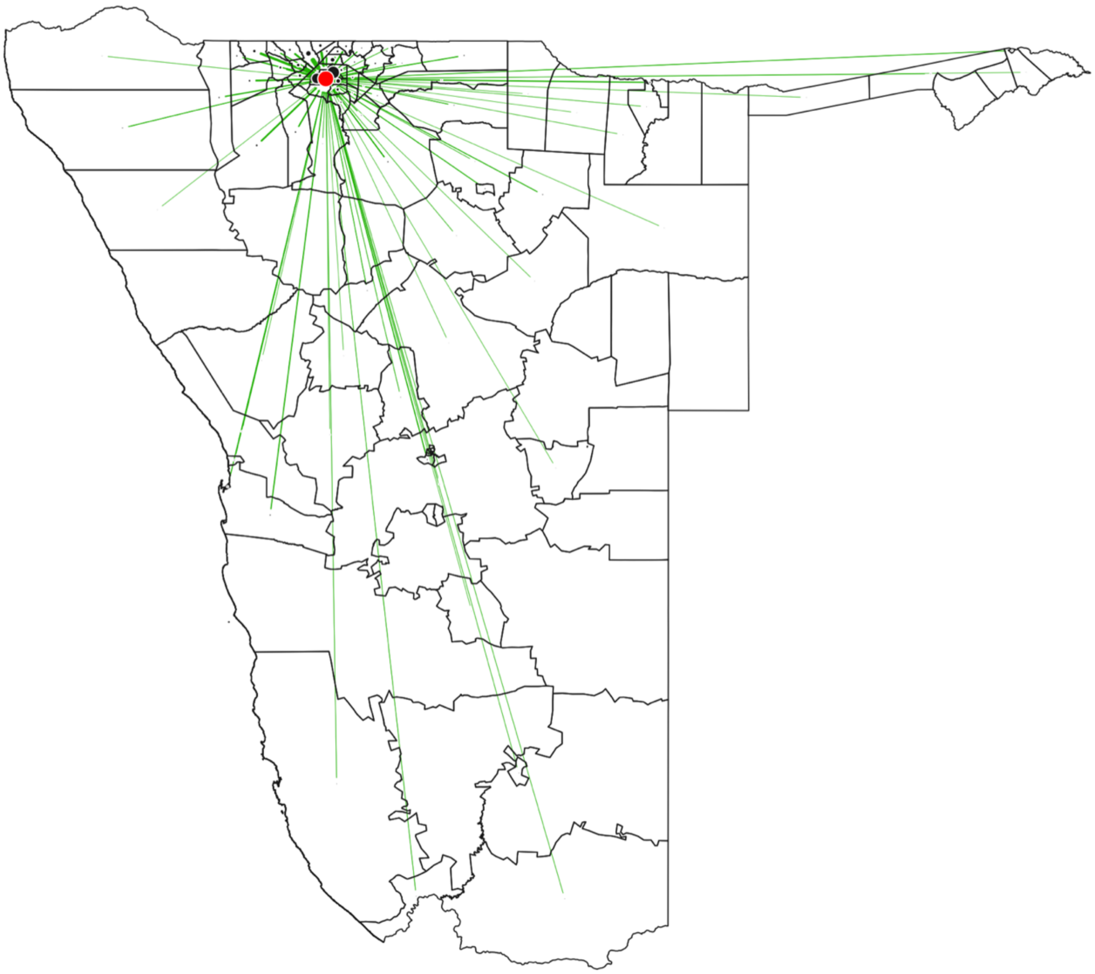

Supplementary Figure 4: Importation of risk. Map showing the in-flow risk flow network for men; risk is due to HIV-infected female residents of other constituencies visiting Oshakati East (represented by the red dot). The thickness of the green lines is proportional to the amount of imported risk.

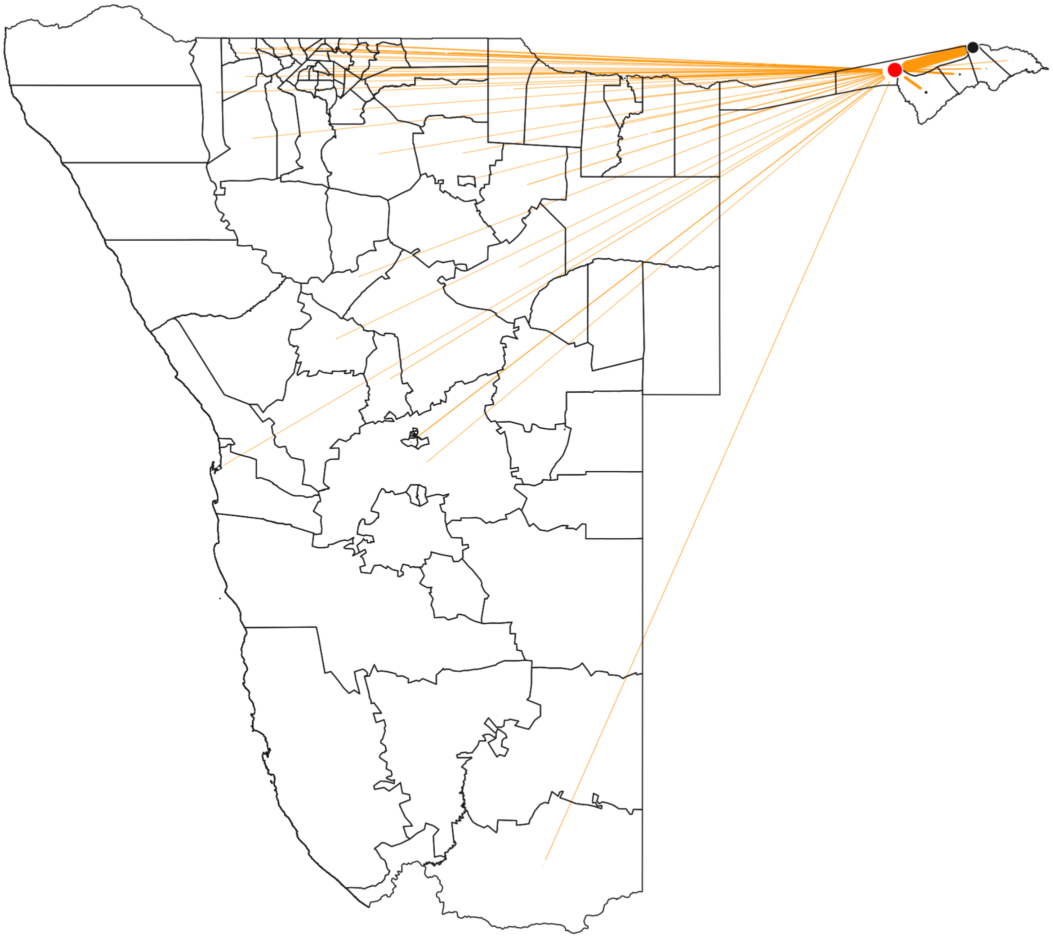

Supplementary Figure 5: Importation of risk. Map showing the in-flow risk flow network for men; risk is due to uninfected male residents of Kongola (represented by the red dot) visiting other constituencies. The thickness of the orange lines is proportional to the amount of imported risk; the largest amount of imported risk is from Katima Muliro Urban (black dot).

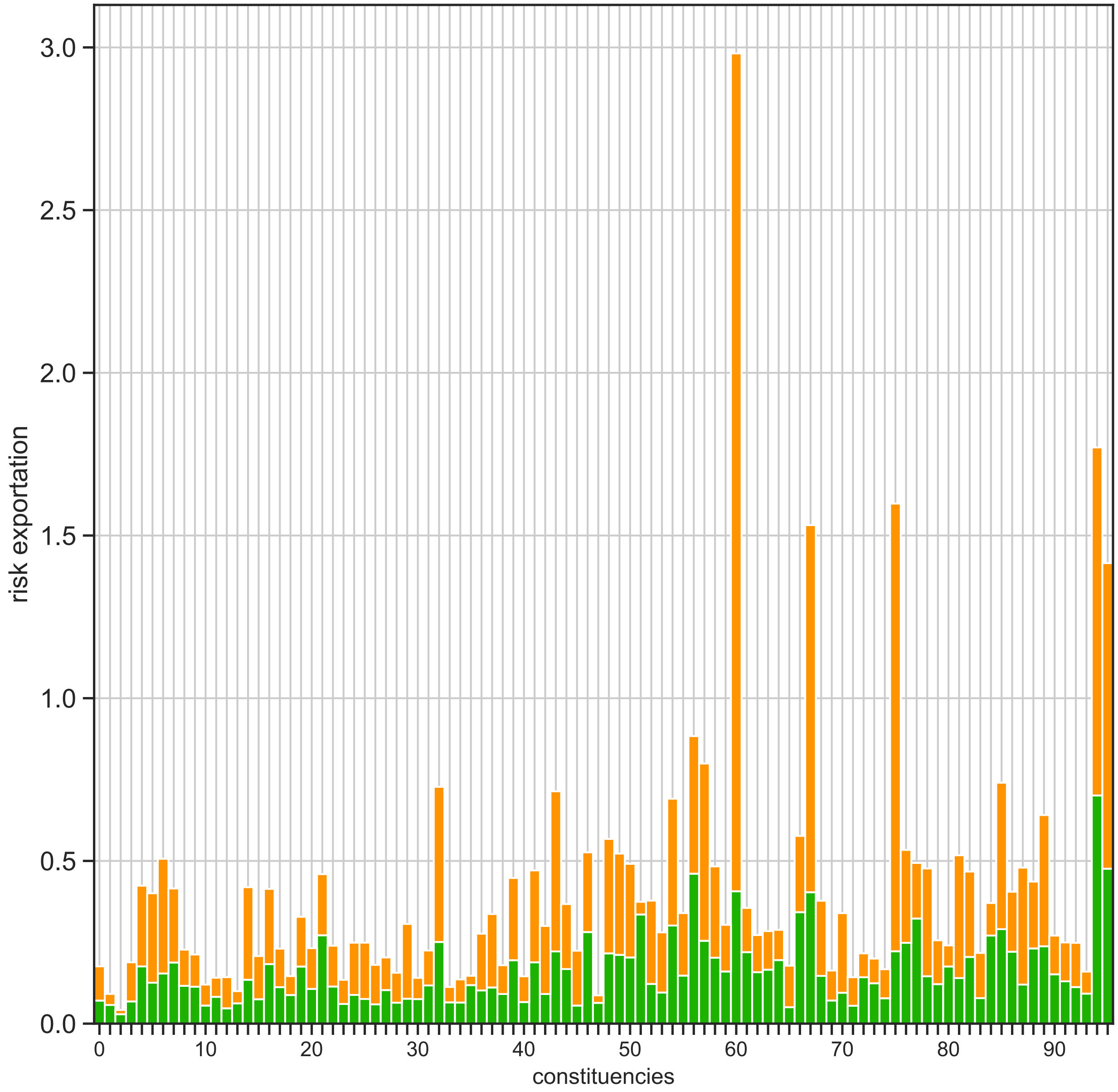

Supplementary Figure 6: Exportation of risk. Histogram showing the exportation of risk for women from each constituency. The y-axis shows the value for exported risk; a mathematical definition of exported risk is given in equation (5). The numbers on the x-axis refer to specific constituencies; the key code is given in Supplementary Table 3. Constituencies are ordered as in Supplementary Fig. 3. Orange data show the risk for women that was exported from each constituency due to visits from uninfected women who lived in other constituencies. Green data show the risk for women that was exported from each constituency due to travel by their HIV-infected male residents. The stacked value represents the total amount of exported risk.

Supplementary Table 1: Frequency of usage of mobile phones in Namibia in 2012 stratified by gender. Data are from the 5th round of Afrobarometer surveys[1]. Survey respondents were asked how often they used a mobile phone for calls and texting. Frequency of phone usage was very similar between men and women.

Number of times per day		≥ 5	3–4	1–2	< 1♦	Never
Phone calls*	Men	45%	17%	17%	12%	8%
Phone calls	Women	43%	15%	20%	14%	8%
Texting	Men	60%	13%	9%	6%	12%
Texting*	Women	57%	13%	9%	9%	11%

♦Uses phone on average less than 1 time per day.
*Percentages do not add up to 100% due to rounding.

Supplementary Table 2: Travel data from the 2013 Demographic Health Survey in Namibia[2]. Data are stratified by gender, and represent individuals aged 15 to 49 years old. Participants in the survey were asked how many overnight trips they had taken in the previous 12 months, and whether or not they had been away for more than one month.

Took an overnight trip (previous 12 months)	women	men
No	5,681 (63%)	2,205 (57%)
Yes	3,488 (37%)	1,722 (43%)
Total	9,169	3,927
**Amount of time away (previous 12 months)**	**women**	**men**
> 1 month	1,687 (18%)	717 (18%)
< 1 month	1,801 (19%)	1,005 (25%)
not away	5,681 (63%)	2,205 (57%)
Total	9,169	3,927

Supplementary Table 3: Calculated risks for women: local risk (i.e., from HIV-infected male residents of their home constituency), imported risk, and exported risk. Constituencies are sorted by increasing prevalence of HIV in men. *t*-risk can be imported (equation (12)) or exported (equation (14)). *v*-risk can be imported (equation (13)) or exported (equation (15)). Local risk is calculated using equation (6). The index for each constituency corresponds to the numbers shown on the x-axis in Supplementary Fig. 3 and Supplementary Fig. 6.

index	constituency	local	v import	t import	v export	t export
0	Tsumeb	0.0272	0.0127	0.0190	0.0708	0.1052
1	Daures	0.0268	0.0083	0.0225	0.0573	0.0339
2	Tsumkwe	0.0388	0.0030	0.0144	0.0290	0.0130
3	Omatako	0.0297	0.0158	0.0209	0.0681	0.1202
4	Engela	0.0307	0.0148	0.0236	0.1758	0.2480
5	Oshikango	0.0345	0.0170	0.0193	0.1258	0.2748
6	Eenhana	0.0318	0.0200	0.0225	0.1541	0.3525
7	Ohangwena	0.0312	0.0143	0.0258	0.1878	0.2275
8	Okakarara	0.0477	0.0083	0.0164	0.1162	0.1112
9	Steinhausen	0.0427	0.0095	0.0216	0.1134	0.0989
10	Epukiro	0.0440	0.0088	0.0204	0.0555	0.0644
11	Aminius	0.0495	0.0059	0.0170	0.0823	0.0588
12	Rehoboth West	0.0452	0.0139	0.0185	0.0470	0.0957
13	Otjombinde	0.0483	0.0050	0.0185	0.0623	0.0375
14	Ondobe	0.0375	0.0176	0.0183	0.1349	0.2844
15	Kalahari	0.0381	0.0174	0.0238	0.0750	0.1326
16	Endola	0.0361	0.0162	0.0277	0.1828	0.2312
17	Ongenga	0.0430	0.0129	0.0252	0.1120	0.1183
18	Omundaungilo	0.0462	0.0071	0.0199	0.0878	0.0582
19	Okongo	0.0458	0.0113	0.0194	0.1754	0.1529
20	Epembe	0.0403	0.0135	0.0252	0.1067	0.1253
21	Omulonga	0.0336	0.0135	0.0335	0.2715	0.1878
22	Mariental Urban	0.0531	0.0097	0.0157	0.1140	0.1255
23	Otjinene	0.0542	0.0084	0.0168	0.0604	0.0742
24	Otavi	0.0461	0.0189	0.0206	0.0882	0.1605
25	Karibib	0.0510	0.0168	0.0171	0.0757	0.1730
26	Omaruru	0.0539	0.0143	0.0166	0.0594	0.1212
27	Oshikuku	0.0477	0.0167	0.0336	0.1031	0.1002
28	Gibeon	0.0539	0.0147	0.0196	0.0643	0.0920
29	Mariental Rural	0.0398	0.0251	0.0195	0.0765	0.2298
30	Rehoboth Rural	0.0633	0.0095	0.0178	0.0754	0.0653
31	Berseba	0.0611	0.0136	0.0204	0.1173	0.1073

Continuation of Supplementary Table 3.

index	constituency	local	v import	t import	v export	t export
32	Gobabis	0.0613	0.0130	0.0162	0.2509	0.4769
33	Keetmanshoop Rural	0.0679	0.0090	0.0179	0.0653	0.0473
34	Oranjemund	0.0618	0.0118	0.0239	0.0648	0.0710
35	Epupa	0.0706	0.0040	0.0222	0.1184	0.0286
36	Kamanjab	0.0565	0.0186	0.0232	0.1020	0.1744
37	Karas	0.0601	0.0215	0.0181	0.1111	0.2257
38	Sesfontein	0.0589	0.0150	0.0239	0.0910	0.0884
39	Keetmanshoop Urban	0.0661	0.0128	0.0180	0.1949	0.2527
40	Luderitz	0.0747	0.0092	0.0134	0.0663	0.0791
41	Grootfontein	0.0629	0.0149	0.0202	0.1883	0.2825
42	Ruacana	0.0561	0.0238	0.0223	0.0910	0.2090
43	Outapi	0.0618	0.0233	0.0203	0.2219	0.4919
44	Okaku	0.0489	0.0196	0.0333	0.1677	0.1997
45	Okatyali	0.0244	0.0351	0.0462	0.0555	0.1686
46	Uukwiyu	0.0473	0.0147	0.0437	0.2814	0.2450
47	Uuvudhiya	0.0633	0.0074	0.0317	0.0638	0.0231
48	Omuthiyagwipundi	0.0654	0.0185	0.0223	0.2159	0.3516
49	Opuwo	0.0764	0.0135	0.0152	0.2113	0.3112
50	Windhoek Rural	0.0641	0.0160	0.0243	0.2033	0.2877
51	Walvisbay Rural	0.0624	0.0034	0.0434	0.3354	0.0389
52	Okahao	0.0676	0.0239	0.0212	0.1219	0.2561
53	Arandis	0.0574	0.0277	0.0215	0.0956	0.1848
54	Otjiwarongo	0.0769	0.0128	0.0171	0.3014	0.3897
55	Khorixas	0.0790	0.0117	0.0168	0.1475	0.1921
56	Swakopmund	0.0780	0.0120	0.0194	0.4607	0.4228
57	Walvisbay Urban	0.0703	0.0230	0.0165	0.2545	0.5448
58	Okahandja	0.0773	0.0133	0.0181	0.2025	0.2807
59	Anamulenge	0.0596	0.0178	0.0371	0.1602	0.1433
60	Windhoek West	0.0574	0.0370	0.0134	0.4068	2.5747
61	Kahenge	0.0839	0.0105	0.0250	0.2197	0.1358
62	Mukwe	0.0936	0.0084	0.0167	0.1581	0.1141
63	Kapako	0.0718	0.0164	0.0370	0.1658	0.1186

Continuation of Supplementary Table 3.

index	constituency	local	v import	t import	v export	t export
64	Mashare	0.0768	0.0112	0.0350	0.1952	0.0929
65	Ndiyona	0.0740	0.0264	0.0198	0.0504	0.1275
66	Ongwediva	0.0678	0.0104	0.0330	0.3425	0.2343
67	Ondangwa	0.0538	0.0311	0.0257	0.4038	1.1281
68	Outjo	0.0784	0.0147	0.0183	0.1467	0.2309
69	Kongola	0.0693	0.0248	0.0396	0.0710	0.0921
70	Katima Muliro Rural	0.0627	0.0356	0.0345	0.0948	0.2447
71	Linyandi	0.0780	0.0232	0.0313	0.0549	0.0876
72	Kabe	0.0807	0.0115	0.0397	0.1425	0.0735
73	Sibinda	0.0805	0.0133	0.0378	0.1240	0.0759
74	Mpungu	0.0861	0.0165	0.0183	0.0779	0.0892
75	Oshakati East	0.0440	0.0460	0.0211	0.2222	1.3759
76	Onyaanya	0.0692	0.0170	0.0295	0.2483	0.2855
77	Oniipa	0.0658	0.0094	0.0350	0.3230	0.1708
78	Onayena	0.0605	0.0283	0.0261	0.1456	0.3311
79	Olukonda	0.0643	0.0170	0.0342	0.1213	0.1348
80	Okankolo	0.0724	0.0082	0.0365	0.1756	0.0648
81	Omuntele	0.0646	0.0281	0.0228	0.1400	0.3772
82	Engodi	0.0667	0.0192	0.0265	0.2049	0.2624
83	Guinas	0.0562	0.0247	0.0246	0.0783	0.1390
84	Oshakati West	0.0722	0.0064	0.0397	0.2707	0.1000
85	Etayi	0.0737	0.0174	0.0228	0.2903	0.4500
86	Tsandi	0.0877	0.0112	0.0228	0.2210	0.1843
87	Elim	0.0590	0.0343	0.0263	0.1201	0.3593
88	Ogongo	0.0670	0.0168	0.0360	0.2311	0.2054
89	Okalongo	0.0735	0.0210	0.0246	0.2377	0.4032
90	Onesi	0.0799	0.0134	0.0269	0.1516	0.1188
91	Otamanzi	0.0824	0.0144	0.0257	0.1300	0.1194
92	Rundu Rural East	0.0828	0.0254	0.0388	0.1124	0.1359
93	Rundu Rural West	0.0964	0.0197	0.0364	0.0921	0.0677
94	Rundu Urban	0.1203	0.0194	0.0187	0.7011	1.0696
95	Katima Muliro Urban	0.1535	0.0232	0.0184	0.4760	0.9392

Supplementary Table 4: Calculated risks for men: local risk (i.e., from HIV-infected female residents of their home constituency), imported risk, and exported risk. Constituencies are sorted by increasing prevalence in women. *t*-risk can be imported (equation (12)) or exported (equation (14)). *v*-risk can be imported (equation (13)) or exported (equation (15)). Local risk is calculated using equation (6). The index for each constituency corresponds to the numbers shown on the x-axis in Fig. 3a and Fig. 4a.

index	constituency	local	v import	t import	v export	t export
0	Steinhausen	0.0369	0.0103	0.0255	0.0914	0.0853
1	Otjombinde	0.0417	0.0055	0.0216	0.0496	0.0305
2	Epukiro	0.0380	0.0091	0.0239	0.0455	0.0558
3	Aminius	0.0427	0.0066	0.0205	0.0622	0.0458
4	Kalahari	0.0332	0.0199	0.0285	0.0602	0.1175
5	Otjinene	0.0465	0.0100	0.0212	0.0462	0.0614
6	Rehoboth West	0.0514	0.0190	0.0247	0.0389	0.0822
7	Gobabis	0.0534	0.0147	0.0209	0.1990	0.4178
8	Mariental Urban	0.0627	0.0143	0.0235	0.0924	0.1048
9	Epupa	0.0646	0.0042	0.0269	0.0923	0.0238
10	Kamanjab	0.0517	0.0228	0.0283	0.0797	0.1384
11	Sesfontein	0.0538	0.0158	0.0277	0.0746	0.0738
12	Opuwo	0.0628	0.0167	0.0230	0.1385	0.2447
13	Khorixas	0.0629	0.0159	0.0228	0.0881	0.1226
14	Outjo	0.0610	0.0203	0.0255	0.0861	0.1488
15	Mariental Rural	0.0510	0.0541	0.0346	0.0626	0.2125
16	Gibeon	0.0698	0.0210	0.0280	0.0579	0.0834
17	Tsumkwe	0.0932	0.0041	0.0208	0.0546	0.0265
18	Omatako	0.0713	0.0228	0.0299	0.1179	0.1785
19	Rehoboth Rural	0.0844	0.0128	0.0241	0.0759	0.0648
20	Okakarara	0.0895	0.0119	0.0241	0.1532	0.1512
21	Windhoek West	0.0604	0.0588	0.0216	0.2996	2.2944
22	Windhoek Rural	0.0730	0.0222	0.0342	0.1677	0.2521
23	Daures	0.0859	0.0110	0.0321	0.1286	0.0695
24	Otavi	0.0767	0.0307	0.0336	0.0925	0.1688
25	Tsumeb	0.0922	0.0205	0.0307	0.1484	0.1816
26	Grootfontein	0.0904	0.0248	0.0336	0.1615	0.2449
27	Karibib	0.0916	0.0260	0.0252	0.0881	0.1812
28	Omaruru	0.0927	0.0240	0.0257	0.0637	0.1194
29	Keetmanshoop Urban	0.0984	0.0204	0.0279	0.1872	0.2415
30	Luderitz	0.1129	0.0156	0.0224	0.0607	0.0734
31	Otjiwarongo	0.1002	0.0196	0.0263	0.2644	0.3525

Continuation of Supplementary Table 4.

index	constituency	local	v import	t import	v export	t export
32	Arandis	0.0767	0.0390	0.0327	0.0812	0.1673
33	Swakopmund	0.1015	0.0184	0.0299	0.3941	0.3695
34	Okahandja	0.0997	0.0210	0.0278	0.1720	0.2429
35	Walvisbay Rural	0.0834	0.0045	0.0640	0.3011	0.0366
36	Walvisbay Urban	0.0915	0.0357	0.0267	0.2098	0.4739
37	Oranjemund	0.1032	0.0190	0.0361	0.0700	0.0743
38	Karas	0.1023	0.0347	0.0293	0.1199	0.2404
39	Keetmanshoop Rural	0.1163	0.0138	0.0277	0.0729	0.0525
40	Berseba	0.1084	0.0192	0.0296	0.1428	0.1258
41	Omuthiyagwipundi	0.1018	0.0311	0.0369	0.2021	0.3298
42	Ndiyona	0.1091	0.0390	0.0295	0.0499	0.1270
43	Mukwe	0.1380	0.0131	0.0259	0.1480	0.1064
44	Kahenge	0.1237	0.0159	0.0381	0.2110	0.1324
45	Kapako	0.1058	0.0247	0.0562	0.1595	0.1162
46	Mashare	0.1132	0.0166	0.0518	0.1939	0.0922
47	Okankolo	0.1037	0.0126	0.0570	0.1584	0.0576
48	Guinas	0.0805	0.0437	0.0447	0.0611	0.1196
49	Omuntele	0.0926	0.0442	0.0366	0.1230	0.3419
50	Engodi	0.0956	0.0338	0.0463	0.1679	0.2255
51	Onayena	0.0868	0.0452	0.0438	0.1224	0.2911
52	Onyaanya	0.0992	0.0275	0.0477	0.2135	0.2499
53	Oniipa	0.0944	0.0219	0.0669	0.2441	0.1253
54	Olukonda	0.0921	0.0307	0.0585	0.0999	0.1118
55	Mpungu	0.1259	0.0255	0.0299	0.0646	0.0824
56	Oshakati West	0.1097	0.0112	0.0693	0.2312	0.0858
57	Oshakati East	0.0703	0.0845	0.0387	0.1930	1.2825
58	Ondangwa	0.0896	0.0575	0.0461	0.3770	1.0657
59	Ongwediva	0.1130	0.0214	0.0625	0.2964	0.2041
60	Engela	0.1088	0.0419	0.0611	0.2474	0.3239
61	Elim	0.1008	0.0598	0.0466	0.1154	0.3525
62	Ogongo	0.1146	0.0316	0.0657	0.2173	0.1968
63	Okalongo	0.1256	0.0395	0.0451	0.2245	0.3909

Continuation of Supplementary Table 4.

index	constituency	local	v import	t import	v export	t export
64	Onesi	0.1365	0.0250	0.0512	0.1407	0.1170
65	Otamanzi	0.1408	0.0245	0.0448	0.1274	0.1189
66	Tsandi	0.1499	0.0208	0.0423	0.2086	0.1799
67	Oshikango	0.1206	0.0447	0.0450	0.1906	0.3655
68	Etayi	0.1285	0.0378	0.0452	0.2526	0.4018
69	Eenhana	0.1099	0.0508	0.0503	0.2337	0.4576
70	Ohangwena	0.1063	0.0393	0.0663	0.2567	0.2943
71	Anamulenge	0.1155	0.0317	0.0672	0.1739	0.1558
72	Okahao	0.1364	0.0408	0.0363	0.1434	0.2900
73	Ondobe	0.1213	0.0478	0.0451	0.1817	0.3669
74	Omulonga	0.1076	0.0280	0.0760	0.3869	0.2585
75	Endola	0.1156	0.0370	0.0610	0.2606	0.3048
76	Ongenga	0.1376	0.0274	0.0511	0.1696	0.1604
77	Omundaungilo	0.1479	0.0195	0.0494	0.1185	0.0757
78	Okongo	0.1467	0.0238	0.0383	0.3038	0.2282
79	Epembe	0.1289	0.0303	0.0526	0.1570	0.1618
80	Outapi	0.1390	0.0407	0.0354	0.2850	0.6022
81	Ruacana	0.1295	0.0381	0.0381	0.1355	0.3065
82	Rundu Rural East	0.1213	0.0374	0.0573	0.1106	0.1345
83	Oshikuku	0.1306	0.0288	0.0590	0.1605	0.1421
84	Rundu Rural West	0.1408	0.0291	0.0546	0.0884	0.0665
85	Okaku	0.1167	0.0372	0.0631	0.2075	0.2317
86	Uuvudhiya	0.1510	0.0092	0.0434	0.1271	0.0444
87	Uukwiyu	0.1129	0.0262	0.0795	0.3539	0.2886
88	Okatyali	0.0582	0.0727	0.0875	0.0702	0.1906
89	Rundu Urban	0.1756	0.0294	0.0288	0.6660	1.0338
90	Sibinda	0.1794	0.0248	0.0695	0.1428	0.0853
91	Linyandi	0.1738	0.0428	0.0561	0.0652	0.0992
92	Kongola	0.1544	0.0452	0.0694	0.0864	0.1030
93	Katima Muliro Rural	0.1398	0.0660	0.0608	0.1181	0.2766
94	Kabe	0.1799	0.0215	0.0733	0.1669	0.0849
95	Katima Muliro Urban	0.2526	0.0481	0.0353	0.4155	0.8213

Supplementary References:

1 Afrobarometer Data. *Namibia round 5, 2012*. http://www.afrobarometer.org/ (accessed Oct 26, 2020).

2 Ministry of Health and Social Services & Namibia Statistics Agency. *Namibia Demographic and Health Survey 2013*. https://dhsprogram.com/pubs/pdf/FR298/FR298.pdf (Ministry of Health and Social Services, and Namibia Statistics Agency, 2014).
