# Supplementary material for: Using mobile phone data to reveal risk flow networks underlying the HIV epidemic in Namibia
Source: Nat Commun. 2021 May 14;12:2837. doi: 10.1038/s41467-021-23051-w (PMC8121904; doi:10.1038/s41467-021-23051-w)
Supplement: Supplementary file 3 — Reporting Summary [file 41467_2021_23051_MOESM3_ESM.pdf]

nature research

Corresponding author(s): Sally Blower

Last updated by author(s): Mar 31, 2021

Reporting Summary

Nature Research wishes to improve the reproducibility of the work that we publish. This form provides structure for consistency and transparency in reporting. For further information on Nature Research policies, see our Editorial Policies and the Editorial Policy Checklist.

Statistics

For all statistical analyses, confirm that the following items are present in the figure legend, table legend, main text, or Methods section.

n/a	Confirmed	
[ ]	[x]	The exact sample size (*n*) for each experimental group/condition, given as a discrete number and unit of measurement
[x]	[ ]	A statement on whether measurements were taken from distinct samples or whether the same sample was measured repeatedly
[x]	[ ]	The statistical test(s) used AND whether they are one- or two-sided<br>*Only common tests should be described solely by name; describe more complex techniques in the Methods section.*
[ ]	[x]	A description of all covariates tested
[x]	[ ]	A description of any assumptions or corrections, such as tests of normality and adjustment for multiple comparisons
[x]	[ ]	A full description of the statistical parameters including central tendency (e.g. means) or other basic estimates (e.g. regression coefficient) AND variation (e.g. standard deviation) or associated estimates of uncertainty (e.g. confidence intervals)
[x]	[ ]	For null hypothesis testing, the test statistic (e.g. *F*, *t*, *r*) with confidence intervals, effect sizes, degrees of freedom and *P* value noted<br>*Give P values as exact values whenever suitable.*
[x]	[ ]	For Bayesian analysis, information on the choice of priors and Markov chain Monte Carlo settings
[x]	[ ]	For hierarchical and complex designs, identification of the appropriate level for tests and full reporting of outcomes
[x]	[ ]	Estimates of effect sizes (e.g. Cohen's *d*, Pearson's *r*), indicating how they were calculated

Our web collection on statistics for biologists contains articles on many of the points above.

Software and code

Policy information about availability of computer code

Data collection	Not applicable as no data was collected.
Data analysis	The gender-specific spatial risk networks were coded in Python 3; analysis of DHS and census data was conducted in R (v. 3.6.3). We now mention the software in the manuscript, and have placed custom code into a public repository (see 'Code availability' statement in manuscript).

For manuscripts utilizing custom algorithms or software that are central to the research but not yet described in published literature, software must be made available to editors and reviewers. We strongly encourage code deposition in a community repository (e.g. GitHub). See the Nature Research guidelines for submitting code & software for further information.

Data

Policy information about availability of data

All manuscripts must include a data availability statement. This statement should provide the following information, where applicable:

- Accession codes, unique identifiers, or web links for publicly available datasets
- A list of figures that have associated raw data
- A description of any restrictions on data availability

We have updated our Data Availability statement as follows:

The 2010-2011 Namibian mobility data in the form of a geographic origin-destination adjacency matrix are freely available at: https://doi.org/10.1371/journal.pcbi.1004846.s002^<ref 34>. The 2013 Namibian Demographic and Health Survey (NDHS) data (Individual recode and HIV test results recode) are available for non-commercial use to registered users at: https://dhsprogram.com/data/dataset/Namibia_Standard-DHS_2013.cfm. The 2011 Namibia Population and Housing census data is freely available for non-commercial use to registered users at: https://nsa.org.na/microdata1/index.php/catalog/19.

Field-specific reporting

Please select the one below that is the best fit for your research. If you are not sure, read the appropriate sections before making your selection.

- [x] Life sciences
- [ ] Behavioural & social sciences
- [ ] Ecological, evolutionary & environmental sciences

For a reference copy of the document with all sections, see nature.com/documents/nr-reporting-summary-flat.pdf

Life sciences study design

All studies must disclose on these points even when the disclosure is negative.

Sample size	We used 3 previously collected datasets: 1) The mobility data consisted of an origin-destination matrix based on a sample of 9 billion texts/calls made from 1.6 million unique SIM cards in Namibia over a period of one year. This has been shown to be sufficiently large in other mobile phone data studies. 2) The NDHS data came from a large nationally-representative sample. This study was also powered for estimation at the subnational (region) level, which is the level we have used it at. 3) The census data was collected for the entire population.
Data exclusions	No data was excluded from this study.
Replication	All code was checked three times; the analysis can be (and was) reproduced.
Randomization	We utilized already collected databases, so randomization was not possible, or relevant. All analyses were conducted at aggregate levels (nothing was done at the individual-level).
Blinding	Blinding is not relevant to study because we are conducting a secondary analysis using non-identifiable data.

Reporting for specific materials, systems and methods

We require information from authors about some types of materials, experimental systems and methods used in many studies. Here, indicate whether each material, system or method listed is relevant to your study. If you are not sure if a list item applies to your research, read the appropriate section before selecting a response.

Materials & experimental systems

n/a	Involved in the study	
x		Antibodies
x		Eukaryotic cell lines
x		Palaeontology and archaeology
x		Animals and other organisms
x		Human research participants
x		Clinical data
x		Dual use research of concern

Methods

n/a	Involved in the study	
x		ChIP-seq
x		Flow cytometry
x		MRI-based neuroimaging
